# P-2209. Hepatitis B Immune Globulin Utilization in Patients Presenting to the Emergency Department Following Sexual Assault

**DOI:** 10.1093/ofid/ofae631.2363

**Published:** 2025-01-29

**Authors:** David E Zimmerman, Megan A Rech, Nicole M Acquisto, Jordan R Covvey, Gavin T Howington, Jordan A Woolum, Giles W Slocum, Tara Flack, Lance Ray, Blake Porter, Vivian Kum, Brett Faine

**Affiliations:** Duquesne University, Pittsburgh, Pennsylvania; Edward Hines Jr. Veterans Affairs Hospital, Hines, Illinois; University of Rochester Medical Center, Rochester, New York; Duquesne University, Pittsburgh, Pennsylvania; University of Kentucky College of Pharmacy/University of Kentucky HealthCare, LEXINGTON, Kentucky; University of Kentucky College of Pharmacy/University of Kentucky HealthCare, LEXINGTON, Kentucky; Rush University Medical Center, Chicago, Illinois; IU Health Methodist Hospital, Indianapolis, Indiana; Denver Health Medical Center, Denver, Colorado; University of Vermont Medical Center, Burlington, Vermont; New York Presbyterian Weill Cornell Medical Center, New York, New York; University of Iowa, Iowa City, Iowa

## Abstract

**Background:**

Sexual assault is an unfortunately common in patients seen in the emergency department (ED). Following sexual assault, patients are at risk of several transmissible infections, including hepatitis B virus (HBV). HBV screening, vaccination, and treatment with hepatitis B immune globulin (HBIG) is recommended in select scenarios by the Centers for Disease Control and Prevention (CDC). HBV vaccine should be administered if the assailant’s HBV status is positive or unknown and the patient has not been previously vaccinated. HBIG administration is recommended if the assailant is known to be HBV positive and the patient has not been previously vaccinated. However, there is a paucity of evidence on compliance with these recommendations in the ED.

**Methods:**

This was a multi-center, retrospective cohort study utilizing the Emergency Medicine Pharmacotherapy Research NETwork (EMPHARM-NET). Patients aged 18 or older were included if they had an ED visit with an ICD-10 diagnosis code corresponding to sexual assault from January 1st, 2021 to January 1st, 2023. The primary endpoint was the number of patients who received HBIG in the ED following sexual assault. Secondary endpoints were the proportion of patients with HBV testing performed and HBV vaccine administration.

**Results:**

A total of 952 patients were included across nine EDs. The HBV status of the assailant(s) was unknown in most patients, 942 (98.9%), negative in 10 (0.1%), and zero confirmed positive assailants. Eight patients (0.8%) received HBIG while in the ED. Regarding HBV vaccination status, 327 (34.3%) patients were previously vaccinated while 108 (11.3%) and 510 (53.5%) did not previously receive or their vaccination status was unknown, respectively. Of the 618 (64.9%) patients who did not have a documented HBV vaccine or the status was unknown, 44 (7.1%) received HBV vaccine while in the ED.

**Conclusion:**

HBIG was rarely administered in the ED due to the difficulty in determining the assailants’ HBV status. The recommendations provided by the CDC guidelines likely result in the restricted adoption of HBIG among sexual assault patients. Opportunities exist to better protect patients affected by sexual assault whose assailants HBV status is unknown and to increase vaccination.

**Disclosures:**

David E. Zimmerman, PharmD, BCCCP, BCEMP, FASHP, AMDA Biologics Inc: Grant/Research Support Nicole M. Acquisto, Pharm.D., FASHP, FCCM, FCCP, BCCCP, Melinda Therapeutics: Grant/Research Support Giles W. Slocum, PharmD, BCCCP, BCEMP, Novo Nordisk: Advisor/Consultant

